# *Dendrobium candidum* aqueous extract attenuates isoproterenol-induced cardiac hypertrophy through the ERK signalling pathway

**DOI:** 10.1080/13880209.2020.1723648

**Published:** 2020-02-17

**Authors:** Yuan-Yuan Cao, Ke Li, Ying Li, Xiao-Ting Tian, Hui-Xue Ba, Aiping Wang, Xiao-Hui Li

**Affiliations:** a Department of Pharmacology, Xiangya School of Pharmaceutical Science, Central South University, Changsha, China; b Hunan Academy of Traditional Chinese Medicine, Hunan, China; c Department of Health Management, The Third Xiangya Hospital, Central South University, Changsha, China; d Hunan Key Laboratory for Bioanalysis of Complex Matrix Samples, Changsha, China; e Institute of Clinical Research, Nanhua Affiliated Hospital, University of South China, Hengyang, China

**Keywords:** Left ventricular systolic pressure, heart-to-body weight ratio, left ventricular/tibia length, extracellular regulated protein kinases, H9c2, phalloidin staining, cardiac fibrosis

## Abstract

**Context:**

The pharmacological functions of *Dendrobium candidum* Wall. ex Lindl. (Orchidaceae) in cardiac hypertrophy remains unclear.

**Objective:**

To evaluate whether *D. candidum* aqueous extract (DCAE) can attenuate experimental cardiac hypertrophy.

**Materials and methods:**

Cardiac hypertrophy in SD rats was induced by subcutaneously injection of isoproterenol (2 mg/kg), once a day for ten days. Rats were gavaged with DCAE (0.13 and 0.78 g/kg) daily for one month. At the end of treatment, measurement of left ventricular systolic pressure (LVSP), heart-to-body weight ratio (HW/BW), left ventricular/tibia length (LV/TL), atrial natriuretic peptide (ANP), brain natriuretic peptide (BNP) levels, haematoxylin-eosin staining, and Masson’s trichrome staining were conducted. In cultured H9c2 cells, DCAE (2 mg/mL) and U0126 (10 μM) were added 2 h before the isoproterenol (10 μM) stimulus. Phalloidin staining was used to evaluate cellular hypertrophy. The mRNA expression of ANP and BNP was measured by qRT-PCR. The expression of p-ERK was determined by immunoblotting.

**Results:**

DCAE treatment significantly reduced the following indicators *in vivo*: (1) the LVSP (16%); (2) HW/BW (13%); (3) LV/TL (6%); (4) ANP (39%); (5) BNP (32%). In cultured H9c2 cells, phalloidin staining showed that DCAE relieved cellular hypertrophy (53% reduction). Furthermore, immunoblotting showed that DCAE can significantly inhibit p-ERK protein expression *in vivo and in vitro* (39% and 27% reduction, respectively).

**Discussion and conclusions:**

DCAE prevents cardiac hypertrophy via ERK signalling pathway and has the potential for treatment of cardiac hypertrophy.

## Introduction

Pathological myocardial hypertrophy is a decompensated response of the myocardial tissue to sustained load increase, which is usually caused by sympathetic nervous excitement and hypertension. Cardiac hypertrophy is also an independent risk factor for ischaemic heart disease, arrhythmia, and sudden cardiac death. Preventing the development of cardiac hypertrophy is conducive to reducing cardiovascular events. The mechanism of cardiac hypertrophy is extremely complicated (Tham et al. 2015). It has been reported that multiple pathogens can activate intracellular signalling transduction pathways, such as the ERK1/2 signalling pathway, leading to a series of biological reactions participating in cardiac hypertrophy (Bernardo et al. 2010). Several medications including angiotensin converting enzyme inhibitors (ACEI) and calcium channel blockers (CCBs) have been used clinically. However, the high mortality rate of patients necessitates more effective therapeutic and protective medications (Tham et al. 2015).

It is historically well-known that Chinese traditional medicine was used in the treatment of cardiovascular diseases. In recent years, studies have shown that herbal extracts play an important role in protecting cardiac function (Tian et al. 2017). For example, luteolin-7-*O*-glucoside pre-treatment had a significant protective effect against doxorubicin-induced cardiotoxicity by reducing intracellular calcium overload and leakage of creatine kinase and lactate dehydrogenase (Yao et al. 2016). Furthermore, some scholars have indicated an ameliorative effect of epigallocatechin gallate on cardiac hypertrophy and fibrosis in aged rats (Muhammed et al. 2018).

*Dendrobium* is a rare Chinese herbal medicine, also known as black stalk. A variety of compounds including flavonoids, saponins, and polysaccharides can be isolated from the dry stem of *D. candidum* Wall. ex Lindl. (Orchidaceae) by employing ion-exchange chromatography, gel filtration chromatography, and high performance liquid chromatography (Tang et al. 2017). Research has revealed the medicinal efficacy of *D. candidum* in cancer, digestive diseases, and immunity diseases. *Dendrobium candidum* has also been shown to have a potent anticancer effect *in vitro* (Zhao et al. 2014), exhibit anti-inflammatory activities, and exert antimetastatic effects (Sun et al. 2016). The polysaccharides within *D. candidum* can improve the proliferative activity of human corneal epithelial cells (HCEC) under a high glucose environment and reduce cell apoptosis by regulating the expression of BAX and BAL-2 (Li et al. 2017). *Dendrobium candidum* also showed preventive effects on dry mouth symptoms and constipation in animal models (Xiao et al. 2011; Wang et al. 2015). Regarding anti-inflammatory effects and immunomodulation, researchers have shown that *D. candidum* has a potential curative effect on lupus nephritis (Wang et al. 2017).

The role of *D. candidum* in cardiac hypertrophy has not been well investigated. Therefore, utilizing the isoproterenol-induced cardiac hypertrophy model in rats and H9c2 cell line, the present study was designed to explore the effect of DCAE on cardiac hypertrophy *in vivo* and *in vitro*.

## Materials and methods

### Preparation of drugs

**Figure 1.**
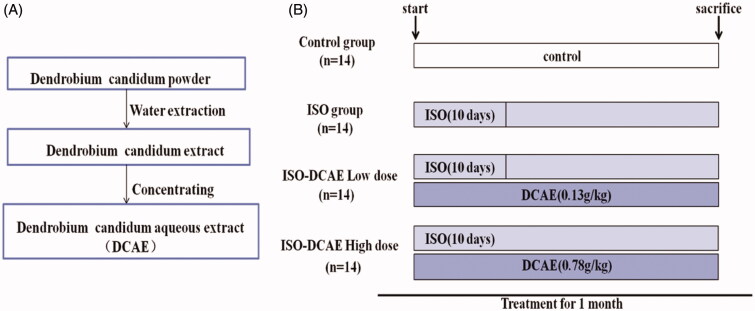
Flow chart of extraction process of *D. candidum* and animal experiment. (A) The aqueous extract product of *D. candidum* is made from the original powder of *D. candidum* (DCAE). (B) Animal model established till the sacrifice process.

Fresh *D. candidum* was purchased from Pingbian County, Yunnan Province, and identified by Li Ruocun of the Hunan Academy of Traditional Chinese Medicine in 2016 (voucher specimen 20160308). The stems of *D. candidum* were pulverized into a fine powder; the powder was decocted in 10 times volume of water twice, first for 2 h and then for 1.5 h. The decoction was collected and filtered, and then the fluids was concentrated to solid extract with a relative density of 1.03 to 1.08 and stored at 4 °C (Figure 1(A)). DCAE (1 g) was extracted from 1.37 g *D. candidum* powder. Polyvinylidene fluoride membranes were purchased from EMD Millipore (Billerica, MA).

### Animals and treatments

The Hunan Normal University Experimental Animal Welfare Ethics Committee and Animal Management and Use Committee approved all animal experimental protocols. Fifty-six healthy male and female Sprague Dawley rats weighing 180–220 g, approximately 6 weeks old, were purchased from SJA Laboratory Animal Co. Ltd (Changsha, Hunan, China). The animals were housed individually in cages under hygienic conditions and placed in a controlled environment with a 12 h light/dark cycle at 24 ± 1 °C and 55 ± 5% humidity for 1 week before the initiation of the experiments. The rats were randomly divided into four groups (14 animals in each group): (1) control group; (2) isoproterenol (ISO) group; (3) ISO + DCAE Low dose (ISO + DCAE L); and (4) ISO + DCAE High dose (ISO + DCAE H). On days 1–10, rats in the ISO group and ISO + DCAE group were subcutaneously injected with isoproterenol (2 mg/kg, Solarbio, Beijing, China) to induce experimental cardiac hypertrophy while rats in the control group were subcutaneously injected with normal saline. At the same time, the DCAE groups were gavaged with DCAE (the corresponding drug contents were low, 0.13 g/kg and high, 0.78 g/kg) (Figure 1(B)).

### Cardiac functional measurements

The animals were sacrificed 30 days after treatment. The rats were anaesthetized using 1% pentobarbital sodium (40 mg/kg). The right carotid artery was separated to allow insertion of a catheter containing 0.05% heparin saline to the left ventricle, and the other end of the catheter was connected to a biological function experiment system (Model: BL-420F, Chengdu Mengtai Technology Co., Ltd.) to record the changes in the left ventricular systolic pressure (LVSP).

### Heart-to-body weight ratio (HW/BW) and left ventricular/tibia length (LV/TL)

After being humanely euthanized by bleeding, precooled saline (4 °C) was infused into the left ventricle until the heart paled. The hearts were rapidly excised and rinsed in cold normal saline. Subsequently, the left ventricles (LV, g) and right ventricles (RV, g) were separated and measured, and the tibias removed and length measured (TL, cm). The final body weight (BW, g) along with the weight of heart (HW, g) were recorded. Calculating the HW/BW (g/g) and LV/TL (g/cm) ratio indicated the extent of left ventricular hypertrophy and reflected cardiac hypertrophy.

### Enzyme-linked immunoassay (ELISA) for atrial natriuretic peptide (ANP) and brain natriuretic peptide (BNP)

After sacrificing the animals 30 days post-treatment, blood was collected for detection of ANP and BNP in an ethylenediaminetetraacetic acid (EDTA)-containing anticoagulation tube. The rat blood plasma supernatant was obtained by centrifugation at 3000 rpm, 4 °C, for 10 min. The blood plasma supernatant was separated and the concentrations of atrial natriuretic peptide (ANP) and brain natriuretic peptide (BNP) were measured using ELISA (Wuhan Boster Biological Technology, Hubei, China) according to the manufacturer’s protocol.

### Histological analysis and cytoskeleton staining

The hearts were harvested, fixed with 4% paraformaldehyde, dehydrated, and embedded in paraffin. Paraffin-embedded hearts were cut transversely into 4–5 μm sections. The sections were stained with haematoxylin and eosin (H&E) to display cell morphology, and with three mixed anionic dyes to show collagen deposition. The cytoskeleton was stained using phalloidin dye (Yeasen, 40735ES75, Shanghai, China) to represent cardiac hypertrophy, which was assessed using the NIS-Elements analysis. NIS-Elements analysis was also used to calculate cardiac cell area *in vivo*. The degree of tissue fibrosis was evaluated by collagen assessment (Ashcroft et al. 1988).

### Cell culture and treatment

The rat cardiac myocyte H9c2 cells were provided by Professor Yuan Hong. H9c2 cells are derived from embryonic rat heart tissue and exhibit many properties of skeletal muscle. These cells have long been used as a cardiac cell model in a vast number of cardiac hypertrophy studies (Yeh et al. 2016; Guan et al. 2017). Briefly, H9c2 cells were cultured in Dulbecco’s Modified Eagle’s Medium (DMEM, Gibco, Carlsbad, CA) supplemented with 10% foetal bovine serum and 100 IU/mL penicillin/streptomycin. The cells were maintained at 37 °C and 5% CO_2_ in a humidified incubator. To induce hypertrophy, the cells were treated with isoproterenol (10 μM) for 24 h (Mao et al. 2016). In order to investigate the effect of DCAE on the blockage of isoproterenol-induced hypertrophy, DCAE (2 mg/mL) and U0126, an ERK pathway inhibitor, (10 μM)-rich medium were added 2 h before isoproterenol administration.

### Cell counting kit-8

The cells were seeded in a 96-well plate to a final concentration of 5000 cells/well and incubated in growth media with varying concentrations of DCAE for 24 and 48 h. The medium was changed to fresh medium containing 10 μL of 2-(2-methoxy-4-nitrophenyl)-3-(4-nitrophenyl)-5-(2,4-disulfophenyl)-2*H*-tetrazole monosodium salt (CCK8, Dojindo, Kyushu, Japan) per 100 μL of medium. After 2 h of incubation at 37 °C, the plates were read at a wavelength of 450 nm using a microplate reader (Thermo, Waltham, MA). Six duplicate wells were used for each treatment.

### qRT-PCR

Total mRNA was extracted from H9c2 cardiomyocytes using TRIZOL reagent (Invitrogen, Carlsbad, CA). According to the manufacturer’s instruction, cDNA was synthesized using oligo (dT) primers with the Transcript or First Strand cDNA Synthesis Kit (Thermo Scientific RevertAid First Strand cDNA Synthesis Kit K1621, THERMO). Selected gene differences were confirmed by qRT-PCR using SYBR green (iTaq™ Universal SYBR^®^ Green Supermix, Biorad). The target gene expression was normalized to GAPDH gene expression. The primers for qRT-PCR are shown in Table 1.

**Table 1.** The primers for qRT-PCR.

Name	Forward primer sequence (5′-3′)	Reverse primer sequence (5′-3′)
ANP	GGGCTTCTTCCTCTTCCTGG	TCTGAGACGGGTTGACTTCC
BNP	TAGCCAGTCTCCAGAACAAT	GAGCCATTTCCTCTGACTTT
GAPDH	GACTTTCTTCTCCCGCAGCC	GTCACAAGAGAAGGCAGCCC

### Western blot analysis

The cells were washed twice with ice-cold phosphate-buffered saline and lysed in ice-cold whole cell extraction buffer containing RIPA (Beyotime Biotechnology, Nanjing, Jiangsu, China), protease inhibitors, and phosphatase inhibitors (Bimake, Houston, TX). The protein concentration was determined using the BCA Protein Assay Kit to ensure the loading amount. The proteins were separated on a 10% SDS polyacrylamide gel and electro-transferred to a polyvinylidene fluoride membrane. After blocking with 5% bovine serum albumin-Tris-HCl buffered saline with Tween, primary antibodies (ERK, CST #4695; p-ERK, CST#4370; and GAPDH, Proteintech 10494-1-AP) were detected using horseradish peroxidase-conjugated anti-rabbit antibodies (Jackson 111-035-008, West Grove, PA) and visualized on the Biorad-5200 Chemiluminescent Imaging System.

### Statistical analysis

The quantitative analysis of the Western blot images was performed by using the Image J software. In brief, the scanned images of Western blot were saved in TIFF format. The quantitative values of the Western blot bands were calculated and analysed. Data were presented as mean ± SEM of at least three independent experiments. Statistical analysis of data was performed by the one-way ANOVA. *p* < 0.05 represents a statistically significant difference.

## Results

### DCAE downregulated LVSP, HW/BW, LV/TL, and plasma levels of ANP and BNP in isoproterenol-treated rats

**Figure 2.**
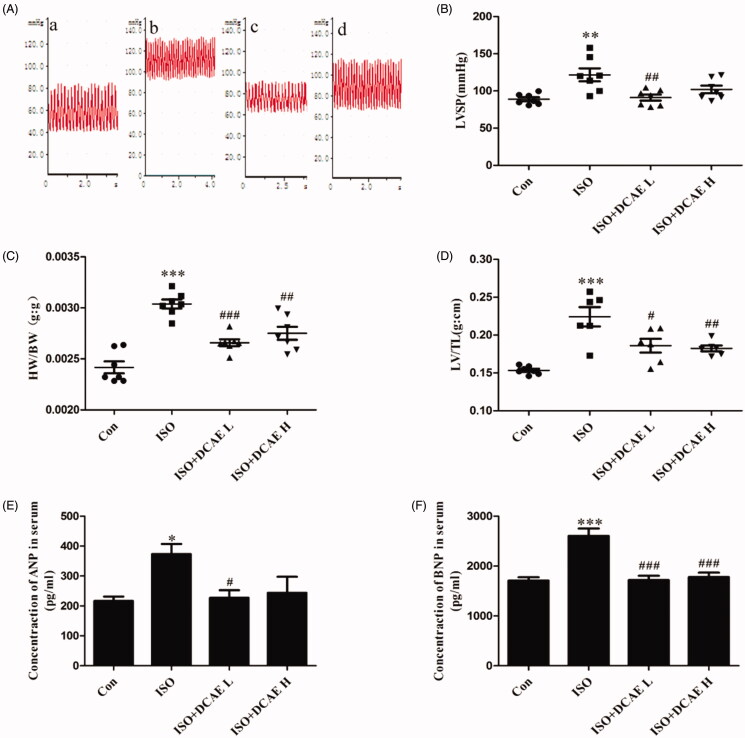
Effects of DCAE on myocardial injury in rats. (A) Vascular blood pressure measurement in rats (a) Control, (b) Isoproterenol, (c) Isoproterenol + DCAE L, (d) Isoproterenol + DCAE H. (B) Vascular blood pressure summary graph (*n* = 7). (C) Heart-to-body weight ratio (HW/BW) summary graph (*n* = 7). (D) Left ventricular weight/tibia length (LV/TL) summary graph (*n* = 7). (E) Atrial natriuretic peptide (ANP) summary graph. (F) Brain natriuretic peptide (BNP) summary graph. Each value shown represents mean ± SEM (*n* = 5). *p < 0.05, **p < 0.01, ***p < 0.001 vs. the control group, #*p* < 0.05, ##*p* < 0.01, ###*p* < 0.001 vs. the isoproterenol group.

Isoproterenol induced a significant increase in LVSP (121.63 ± 3.32 mmHg vs. 88.84 ± 1.02 mmHg, *p* < 0.01), whereas treatment with DCAE (low dose and high dose) attenuated these isoproterenol-induced changes (91.16 ± 1.40 mmHg and 102.04 ± 1.82 mmHg, respectively, *p* < 0.001) (Figure 2(A,B)). HW/BW and LV/TL were significantly higher (0.003 ± 0.000017 g/g vs. 0.0024 ± 0.000022 g/g, 0.22 ± 0.0052 g/cm vs. 0.15 ± 0.00077 g/cm) in the isoproterenol-treated group (*p* < 0.001) than that in the control group. Treatment with DCAE (low dose and high dose) resulted in significant decreases in HW/BW (0.0027 ± 0.000013 g/g and 0.0028 ± 0.000023 g/g, respectively) and LV/TL (0.19 ± 0.0037 g/cm and 0.18 ± 0.0016, g/cm respectively) (*p* < 0.05) (Figure 2(C,D)). DCAE L treatment resulted in decreased levels of ANP (227.29 ± 11.37 pg/mL vs. 373.39 ± 15.15 pg/mL, *p* < 0.05) and BNP (1719.43 ± 34.79 pg/mL vs. 2604.82 ± 59.13 pg/mL, *p* < 0.001) in isoproterenol-induced rats, while DCAE H treatment resulted in a decreased level of BNP (1777.59 ± 36.81 pg/mL, *p* < 0.001) (Figure 2(E,F)).

### DCAE improved cardiac hypertrophy and cardiac fibrosis induced by isoproterenol *in vivo*

**Figure 3.**
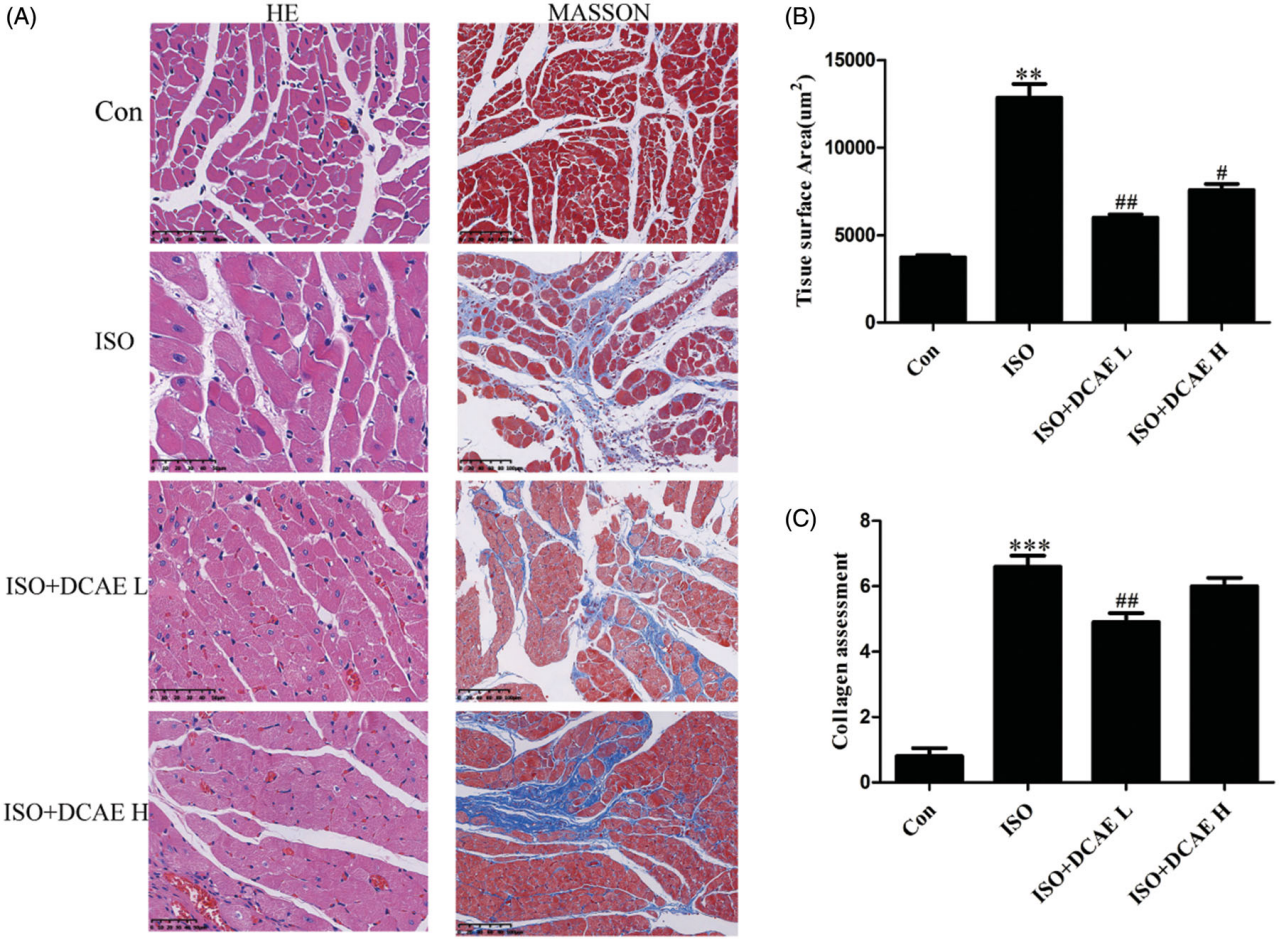
Effects of DCAE on cardiac hypertrophy and cardiac fibrosis *in vivo*. (A) HE (scale bar 50 μm) and Masson (scale bar 100 μm) staining of rat cardiac tissue (*n* = 3). (B) Summary graph of rat myocardial area (*n* = 3). Each value shown represents mean ± SEM. **p < 0.01, ***p < 0.001 vs. the control group. #*p* < 0.05, ##*p* < 0.01 vs. the isoproterenol group.

Heart tissues from isoproterenol-treated rats showed cardiac hypertrophy (12841.12 ± 144.93 μm^2^ vs. 3732.17 ± 977.26 μm^2^, *p* < 0.01), cardiac structural disorders, and cardiac fibrosis when compared to the control group. Treatment with either DCAE L (5994.40 ± 25.23 μm^2^, *p* < 0.01) or DCAE H (7563.60 ± 42.79 μm^2^, *p* < 0.05) produced a marked improvement in isoproterenol-induced cardiac hypertrophy and cardiac fibrosis (Figure 3(A–C)).

### DCAE improved cellular hypertrophy induced by isoproterenol *in vitro*

**Figure 4.**
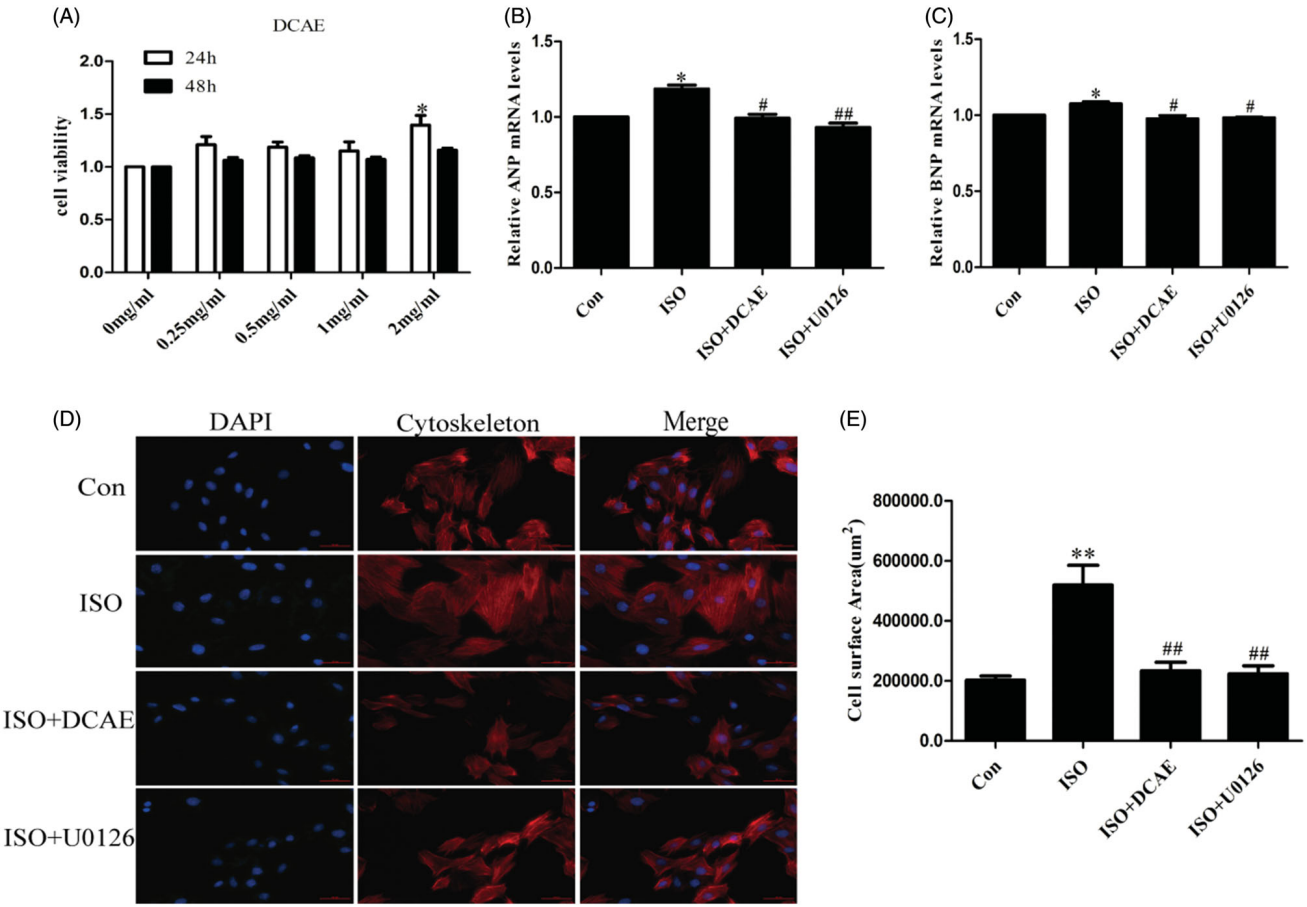
Effects of DCAE-rich medium on cardiomyocytes hypertrophy induced by isoproterenol *in vitro*. (A) The effects of different concentrations of DCAE on the activity of H9c2 cells at different times (*n* = 6). (B) The relative mRNA levels of hypertrophic marker ANP to GAPDH (*n* = 3). (C) The relative mRNA levels of hypertrophic marker BNP to GAPDH (*n* = 3). (D) H9c2 cell phalloidin staining (scale bar 100 μm). (E) Summary graph of H9c2 cells area (*n* = 3). Each value shown represents mean ± SEM. *p < 0.05, **p < 0.01 vs. the control group. #*p* < 0.05, ##*p* < 0.01 vs. the isoproterenol group.

The results of the CCK8 confirmed that DCAE caused no damage to H9c2 cell activity and increased cell viability (*p* < 0.05) (Figure 4(A)). DCAE significantly reduced the upregulated mRNA levels of ANP and BNP induced by isoproterenol (*p* < 0.05) in H9c2 cells (Figure 4(B,C)). Under the induction of isoproterenol, H9c2 cells were obviously hypertrophied as seen with phalloidin staining (519389.38 ± 19967.04 μm^2^ vs. 202013.92 ± 57847.90 μm^2^, *p* < 0.01), but the cell area shrank significantly with pre-treatment of DCAE (233793.05 ± 7619.67 μm^2^, *p* < 0.01). The addition of U0126, an ERK pathway inhibitor, showed a similar effect in limiting hypertrophy (223437.69 ± 7244.15 μm^2^), reinforcing the conclusion that DCAE improves cardiac hypertrophy through inhibition of the ERK pathway (Figure 4(D,E)).

### DCAE suppressed activation of ERK signalling pathways *in vivo* and *in vitro*

**Figure 5.**
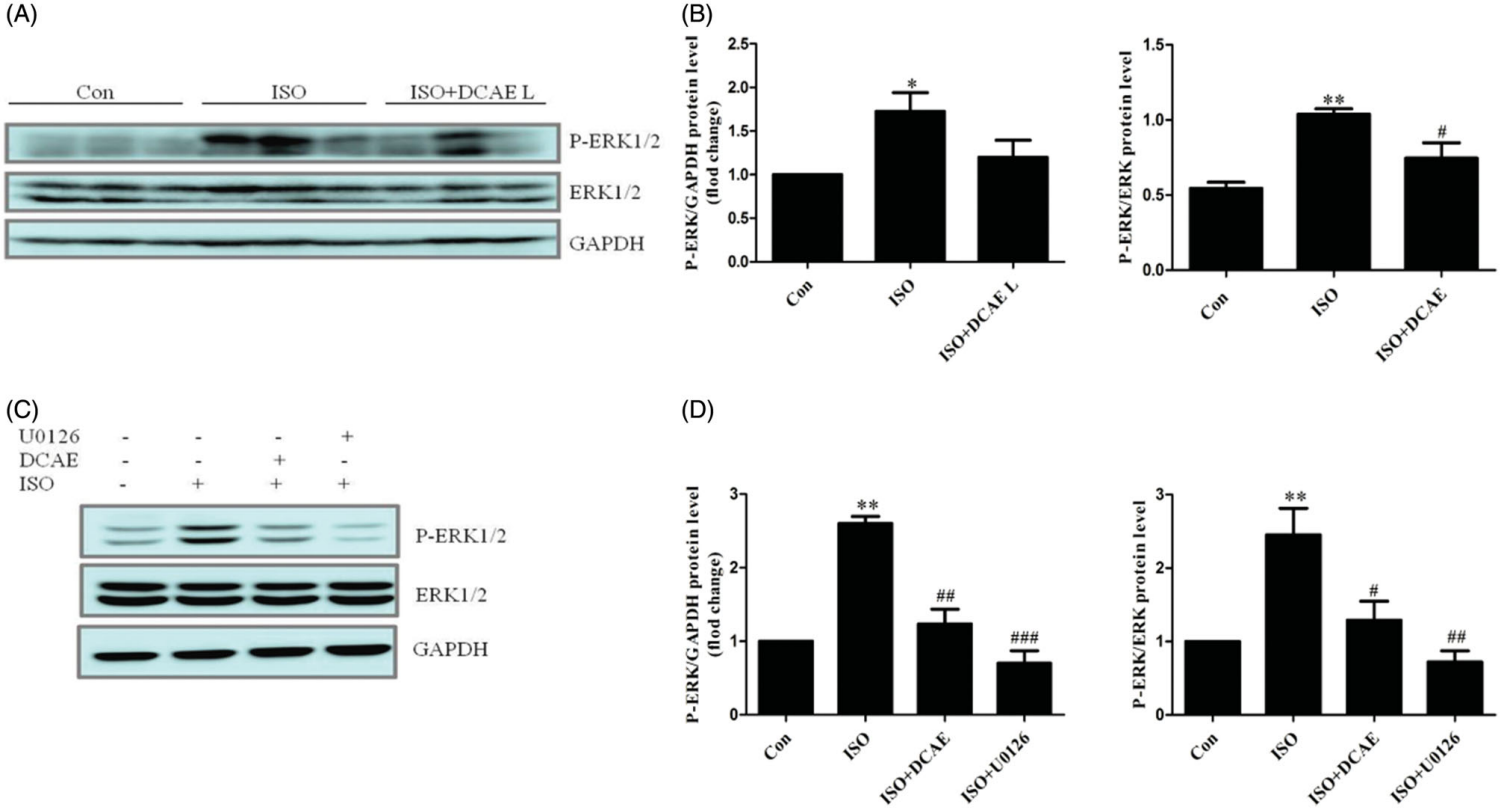
Expression of ERK and p-ERK protein levels *in vivo* and *in vitro*. (A) Effects of DCAE L on p-ERK protein levels in rat cardiac tissue. (B) Summary graph of p-ERK/GAPDH protein levels in rat cardiac tissue (*n* = 3), and summary graph of p-ERK/ERK protein levels in rat cardiac tissue. (C) Effects of DCAE on p-ERK protein levels in H9c2 cells. (D) Summary graph of p-ERK/GAPDH protein levels in H9c2 cells (*n* = 3), and summary graph of p-ERK/ERK protein levels in H9c2 cells (*n* = 3). Each value shown represents mean ± SEM. *p < 0.05, **p < 0.01 vs. the control group. #*p* < 0.05, ##*p* < 0.01, ###*p* < 0.001 vs. the isoproterenol group.

In the rat hearts, isoproterenol injection significantly upregulated the level of p-ERK/ERK (*p* < 0.05). After intervention with DCAE L, the protein level of p-ERK/ERK was decreased (*p* < 0.05) (Figure 5(A,B)). In H9c2 cells, isoproterenol stimulation induced the upregulation of p-ERK/ERK (*p* < 0.01) (Figure 5(C)), which was significantly prevented with DCAE treatment (*p* < 0.01) (Figure 5(D)). U0126, an inhibitor of the ERK/MAPK pathway, also significantly inhibited the phosphorylation of ERK in H9C2 cells incubated with isoproterenol.

## Discussion

The mechanism of cardiac hypertrophy is extremely complicated and thought to be irreversible. However, in recent years, studies have shown that pathological cardiac hypertrophy is indeed reversible under certain conditions (Oka et al. 2014). Along with the management of pathological conditions, including hypertension and congenital heart disease, effective therapeutic medication is a goal for scientists and physicians. Here, we investigated the effects of DCAE on cardiac hypertrophy in rats and cultured H9c2 cells. Results indicated that DCAE significantly improved heart function and cardiac hypertrophy in isoproterenol-induced cardiac hypertrophy models, which may be attributed to inhibition of the ERK signalling pathway.

Hemodynamic data showed that isoproterenol injection in rats caused significant increases in blood pressure, which resulted in impaired cardiac function and an increased post-cardiac load, leading to compensatory cardiac hypertrophy. H&E staining revealed significant cardiac hypertrophy in the isoproterenol-stimulated heart when compared to the control heart. Similarly, phalloidin staining showed that H9c2 cells were swollen under isoproterenol stimulation. Supplementing with DCAE significantly alleviated the phenomenon of cardiac hypertrophy. DCAE reduced LVSP, HW/BW, and LV/TL in isoproterenol-treated rats, and also inhibited myocardial hypertrophy both *in vivo* and *in vitro*. These results strongly suggest that DCAE can improve the hemodynamic indexes and cardiac structure, and thus, protect against cardiac hypertrophy.

Hypertrophy adversely affects normal cardiac function. ANP and BNP are indicated to be markers of myocardial function and play an important role in regulating cell viability. Increased ANP and BNP levels indicate a decline in cardiac function. The results of the present study showed that isoproterenol stimulation significantly up-regulated ANP and BNP expression, whereas DCAE significantly decreased the plasma levels of ANP and BNP in rats with cardiac hypertrophy induced by isoproterenol, which may be attributed to improved myocardial hypertrophy. It is well-known that the renin angiotensin aldosterone system (RAAS) is an important body fluid regulation system and plays an important role in the occurrence and development of left ventricular hypertrophy (Kirby & Johnson 1992). As an agonist of the β-adrenergic receptor (βAR), isoproterenol can activate β1 receptors in the myocardium, causing enhanced myocardial contractility and cardiac output. Long-term over-activation of the myocardial tissue leads to compensatory hypertrophy. Meanwhile, isoproterenol stimulates rennin expression via β2 receptors in the juxtaglomerular cells, causing increased content or activity of aldosterone and Ang II in circulating the blood or tissues, which may finally lead to decompensated hypertrophy. It is known that cardiomyocytes express all three-adrenergic receptor subtypes: β1, β2, and, at least in some species, β3. The β1 subtype is the most prominent and is mainly responsible for the positive chronotropic and inotropic effects of catecholamines. The β2 subtype also increases cardiac function, but its ability to activate non-classical signalling pathways suggests a function distinct from the β1 subtype (Lohse et al. 2003). It was reported that heart can produce Ang II locally; however, this study indicated that Ang II does not mediate isoproterenol-induced cardiac hypertrophy (Golomb et al. 1994). Whether RAAS inhibition was involved in the pharmacological effects of DCAE on cardiac hypertrophy requires further investigation.

The MAPK cascade is an important regulator of cardiomyocyte hypertrophic growth in culture (Bueno et al. 2000). ERK1/2 has been shown to be activated in cultured neonatal rat cardiomyocytes via agonist stimulation and cell stretching (Post et al. 1996; Saucerman et al. 2019). It has been demonstrated that autophosphorylation of ERK1/2 on Thr188 directed ERK1/2 to phosphorylate nuclear targets that are known to cause cardiac hypertrophy (Lorenz et al. 2009; Luan et al. 2015). Several studies have reported that Chinese herbal extracts can improve myocardial function under the stimulation of pathological factors (Sun et al. 2016). Here, we found that DCAE significantly reduced the protein levels of p-ERK, very similar to the effects of U0126, a MEK1-ERK1/2 inhibitor. This data suggests a role for ERK1/2 signalling in the pharmacological effects of DCAE.

In summary, the present study revealed the potential role of *D. candidum* in cardiac hypertrophy treatment. DCAE significantly prevented cardiac hypertrophy induced by isoproterenol both *in vivo* and *in vitro*, which may at least partly be attributed to ERK signalling inhibition. It is worth noting that the low dose of DCAE showed a favourable effect in myocardial protection, and the high dose did not show any additional improvement or any detrimental effects. This result is similar to that of our previous study using *D. candidum* powder, which provides a reference for further research and potential for future clinical use. One of the limitations of this study was that the water extract, rather than the specific ingredients of *D. candidum*, was used in all experiments. The other limitation of this study was that we did not observe the continuous changes after isoproterenol injection and DCAE treatment. Actually, this study was more like a cross-sectional study only describing the changes at one time point, 30 days after treatment. An increase in the BNP and ventricular pressure were both observed in this study, which seemed show different outcome of ventricular failure, the reason may attribute to the progress of heart dysfunction. Systolic and diastolic functions indexes measurements would be helpful to evaluate the real status of heart beside BNP and ventricular pressure. In summary, a whole-process and full-scale investigation will be conducted in the future work. Also, in the future, we hope to reveal the exact ingredients that are effective for treating cardiac hypertrophy in DCAE. Another limitation was that we could not identify the molecular targets of DCAE in cardiac hypertrophy. Aside from the ERK signalling pathway, other underlying mechanisms may be involved in DCAE’s pharmacological function, which require further investigation.

## Conclusions

DCAE alleviated isoproterenol-induced cardiac hypertrophy by regulating the ERK signalling pathway *in vivo* and *in vitro*. However, further research is warranted to determine the exact ingredients in DCAE that are effective for treating cardiac hypertrophy.
